# A right whale (Mysticeti, Balaenidae) from the Pleistocene of Taiwan

**DOI:** 10.1186/s40851-019-0153-z

**Published:** 2019-12-28

**Authors:** Cheng-Hsiu Tsai, Chun-Hsiang Chang

**Affiliations:** 1 Department of Life Science, National Taiwan University, Taipei, 10617 Taiwan; 2 Institute of Ecology and Evolutionary Biology, National Taiwan University, Taipei, 10617 Taiwan; 3 Department of Geology, National Museum of Natural Science, Taichung, 40453 Taiwan

**Keywords:** Cetacea, *Eubalaena*, Fossil, Biogeography, Antitropical distribution, The Pacific Ocean

## Abstract

Current patterns of biological distribution result from the deep past. Of particular interest, some closely related species appear at high latitudes of both hemispheres, but not in between, a pattern known as antitropical distribution. However, the timing, pathway, and drivers of antitropical distributions remain mostly unknown. Here we describe a new fossil, a left tympanic bulla (part of the ear bones), from the Middle/Late Pleistocene (0.78–0.01 mya, but not excluding the possibility of Holocene in age, as the specimen was dredged from the sea bottom and the geological horizon remains uncertain) of Taiwan. The tympanic bulla is diagnostic in baleen whales, and this specimen shows morphological features that are identical to extant *Eubalaena*, including: relatively large size (the anteroposterior length is 117 mm); rectangular outline in medial view; short anterior lobe, judging from the remaining of the lateral furrow; squared anterior margin; prominent transverse crease on the involucrum; transversely compressed in anterior view; well-developed and rounded outer lip; and parallel involucral and main ridges. Although incomplete, the morphological characters and overall similarity to extant *Eubalaena* allow a reliable taxonomic assignment to *Eubalaena* sp. The occurrence of a Pleistocene *Eubalaena* on the southern margin of the western North Pacific is the first balaenid fossil evidence indicative of the biotic interchange between two hemispheres leading to the origin of antitropical distribution in the Pleistocene; alternatively, this specimen might merely represent an extra-limital record of the North Pacific *Eubalaena*. Furthermore, this find suggests that the *Eubalaena* interchange, being one of the largest species displaying antitropical distribution pairs in the history of life, likely took place along the western Pacific. Notably, this does not preclude the *Eubalaena* interchange from other routes, such as the eastern Pacific or the Atlantic Ocean, and future finds should test the scenario for the biotic interchange between Northern and Southern Hemispheres of *Eubalaena*.

## Introduction

Biogeographic patterns are dynamic and constantly evolving. Of various geographical patterns, the antitropical distribution in which closely related species appear at high latitudes of the Northern and Southern Hemispheres, but not in the tropical regions, is singular and remarkable. Recognition of such discontinuous distributions in latitude has been noted since Darwin [1], but the details, including the pathway, timing, and drivers of antitropical distributions remain largely unspecified and speculative [2, 3]. Glacial and interglacial periods during the Pleistocene have been associated with the origins of antitropicality [2, 3]. Yet, in addition to the lack of Pleistocene fossils to support this hypothetical connection, in fact, some dispersal events crossing the equator that gave rise to the antitropical distributions occurred prior to the Pleistocene [4, 5].

**Fig. 1:**
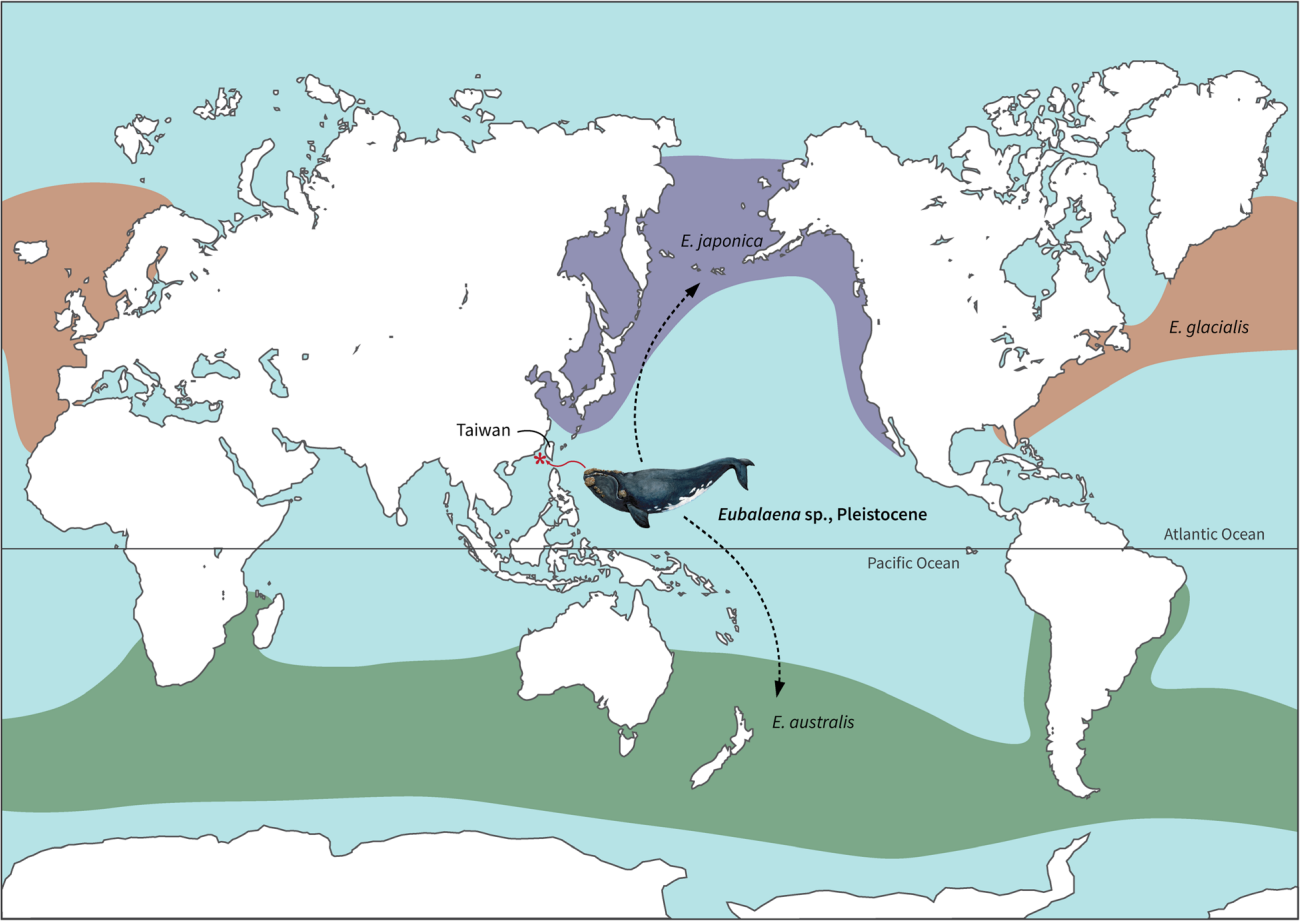
The occurrence of a tropical *Eubalaena* from the Pleistocene of Taiwan (starred) and the proposed distribution of *Eubalaena australis*, *E. glacialis*, and *E. japonica* (tinted, modified from Wilson and Mittermeier [28]). The dash line indicates a possible western Pacific pathway for the *Eubalaena* interchange

Here we describe a new tympanic bulla from the Pleistocene of Taiwan (Fig. 1). The tympanic bulla in Mysticeti (baleen whales) is diagnostic [6–8], and this new specimen matches the unique morphological features of *Eubalaena* (right whales). Given its geological and geographic occurrence, this Pleistocene *Eubalaena* lends support to the glaciation/interglaciation hypothesis. Moreover, it implies that the *Eubalaena* interchange may have taken place between Northern and Southern Hemispheres along the western Pacific (not excluding the possibility of other dispersal routes, such as the eastern Pacific or the Atlantic Ocean). Similarly, this find may support the hypothesis that *E. australis* (Southern right whale) is more closely related to *E. japonica* (North Pacific right whale) than *E. glacialis* (North Atlantic right whale) [9–13]. However, other studies have suggested alternative scenarios for extant *Eubalaena* relationships [14–16], especially a recent, large-scale genomic research substantiating a northern clade with the Southern right whale being the sister taxon [17] that should warrant more considerations and effort to fully reveal the origin and evolutionary history of the antitropical distribution of *Eubalaena*

.

## Materials and methods

Anatomical terms of the tympanic bulla mainly follow Mead and Fordyce [18], Tsai and Fordyce [19], and Tsai and Boessenecker [7], unless specifically stated. Fossil and extant specimens for morphological comparisons are curated in the following collection.

### Institutional abbreviation

**NTUM-VP**: Vertebrate Paleontology, Museum of Zoology, National Taiwan University, Taiwan;

## Results

### Systematic paleontology

Cetacea Brisson, 1762.

Mysticeti Gray, 1864.

Balaenidae Gray, 1821.

*Eubalaena* Gray, 1864.

*Eubalaena* sp.

### Referred specimen

**Fig. 2:**
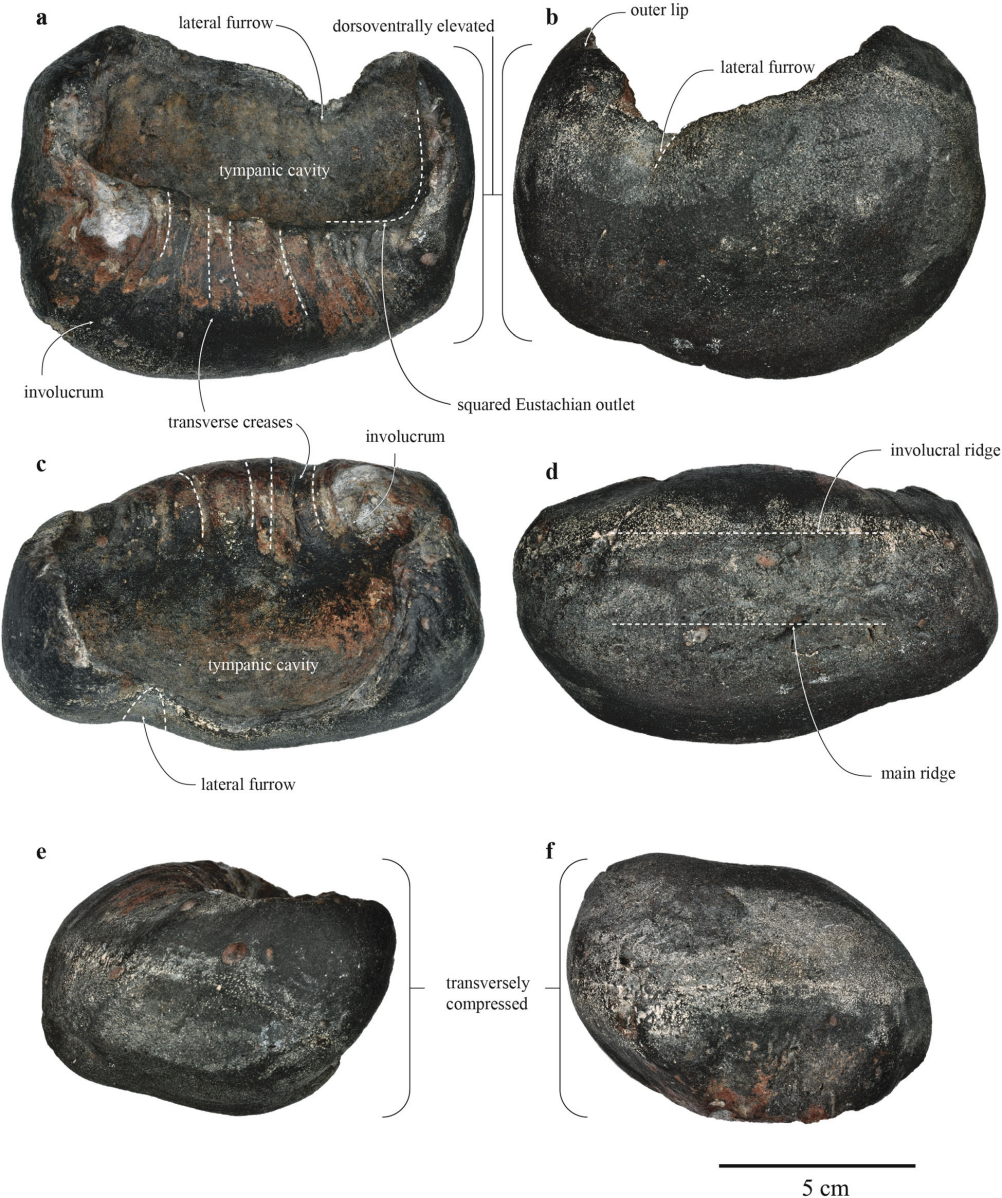
Left tympanic bulla of the Pleistocene *Eubalaena* sp., NTUM-VP 190807. **a** medial view; **b** lateral view; **c** dorsal view; **d** ventral view; **e** anterior view; **f** posterior view

NTUM-VP 190807 is an isolated and incomplete left tympanic bulla (Fig. 2; Table 1). A high-resolution 3D file is digitally curated at doi.org/10.5281/zenodo.3402015 or https://scholars.lib.ntu.edu.tw/handle/123456789/424590 and can be freely downloaded for detailed examination of the morphology.

**Table 1:** Measurements (in mm) of the left tympanic bulla of *Eubalaena* sp., NTUM-VP 190807

Dimension	Measurement (mm)
Maximum length	117.71
Maximum width in dorsal view	65.26
Maximum height,	89.02
Maximum length of the tympanic cavity	86.15
Height of involucrum, from the base of the anterior point of the inner posterior pedicle to ventral-most point	56.94
Length of the anterior lobe, from ventral margin of the lateral furrow to anterior-most tip	36.07

### Locality and geological horizon

Fishermen found the original fossil from the sea bottom during the trawling operations in the Taiwan Strait (widely known as Penghu Channel), and abundant fossils have been recovered over the years. Properly documented fossils from this locality in the paleontological literature are still relatively sparse. However, the faunal composition based on the published fossils includes marine and terrestrial mammals [20–22], reflecting the shallow bed of the Taiwan Strait that converted between sea and land during the interglaciations and glaciations in the Pleistocene. Currently, no evidence is available to identify the precise age of each interglacial marine or glacial terrestrial fossil that was dredged from the Taiwan Strait. Given the disagreement and uncertainty of the geological age among various studies [20–24], for now, this specimen is better to be broadly dated to the Middle to Late Pleistocene (0.78–0.01 mya, but also not excluding the possibility of Holocene in age, as no microfossils were successfully sampled for dating; other attempts, including faunal comparisons and radiometric dating, yielded limited and unclear results) until further research comes to light.

### Diagnosis and descriptive remarks

The dorsal portion of the left tympanic bulla, NTUM-VP 190807, is broken, leaving the tympanic cavity exposed. Regardless of missing some critical features, such as sigmoid and conical processes, broken lateral furrow, and most of the outer lip, the preserved specimen still exhibits distinct and identifiable characters that allow recognition of its taxonomic affinity to *Eubalaena* [6], including (Fig. 2)

1. relatively large size – the anteroposterior length is 117 mm (Table 1);
2. rectangular outline in medial view;
3. short anterior lobe, judging from the remaining of the lateral furrow;
4. dorsally elevated and rounded outer lip on the anterior lobe;
5. squared anterior margin in anterior view;
6. squared Eustachian outlet;
7. prominent transverse creases on the involucrum;
8. transversely compressed in anterior view;
9. parallel involucral and main ridges.

Given the incompleteness of the Taiwan specimen, unresolved morphological differences of earbones (tympanic bulla and periotic) among three extant *Eubalaena* (*E. australis*, *E. glacialis*, and *E. japonica*), and no tympanic bullae of two additional extinct species of *Eubalaena* (*E. ianitrix* [16] and *E. shinshuensis* [25]), this specimen is currently identified as *Eubalaena* sp. In addition, the spongy texture and small size of specimen NTUM-VP 190807 (117 mm) in comparison with the range of *Eubalaena* bullae (112 to 161 mm [[6]]) suggests that it likely belonged to a relatively young individual, complicating further taxonomic assignment to the species level [26, 27]. However, the unique morphological features of *Eubalaena* bullae listed above provide a reliable identification at the genus level.

## Discussion

**Fig. 3:**
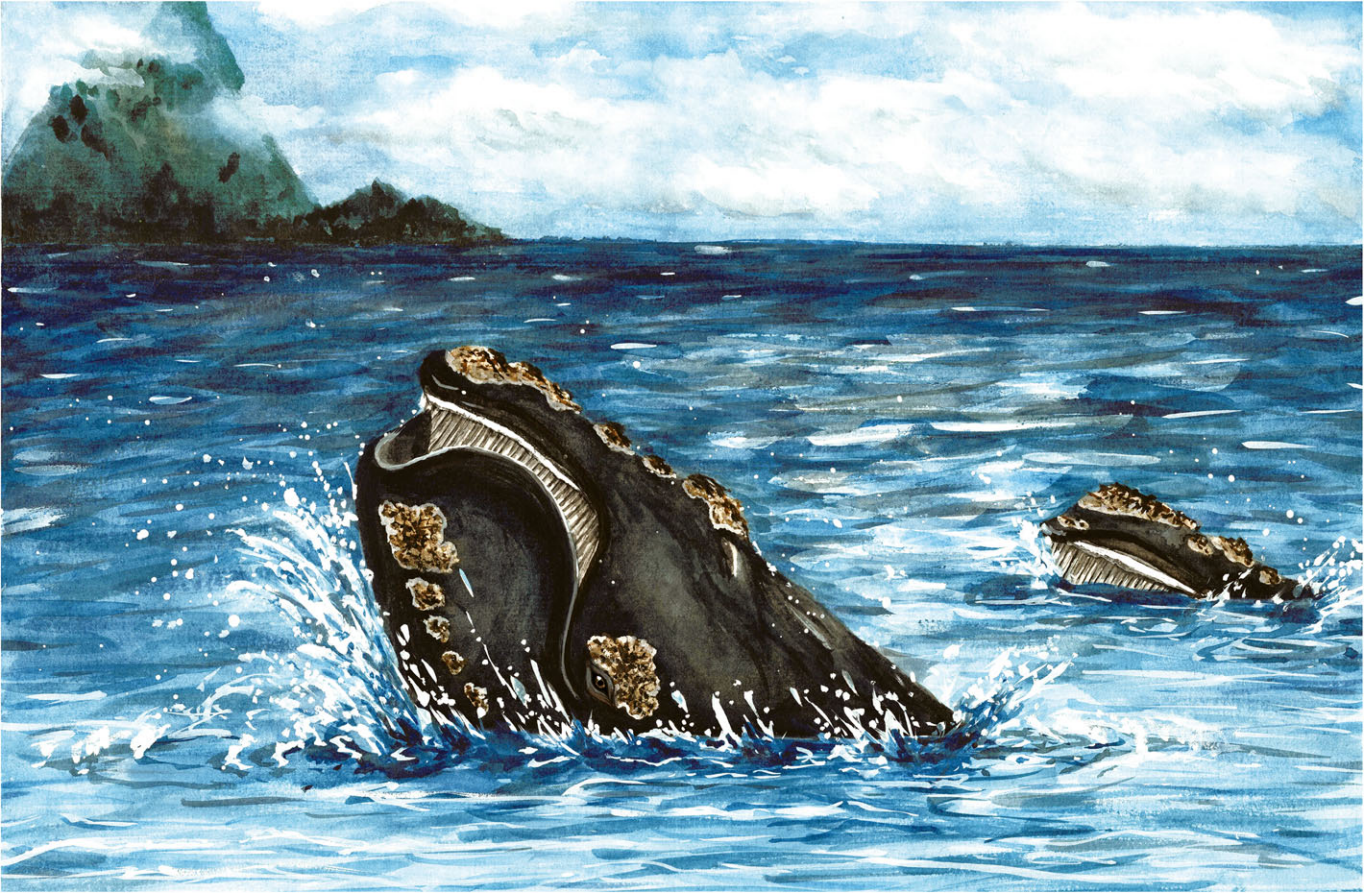
Artistic reconstruction of tropical *Eubalaena* in the Pleistocene of Taiwan (©Lab of Evolution and Diversity of Fossil Vertebrates, National Taiwan University; illustrated by Chang-Han Sun)

The morphology of NTUM-VP 190807, albeit incomplete, broadly matches that of the genus *Eubalaena* as described above (Fig. 2). Taxonomy of extant *Eubalaena* (right whales) remained elusive and inconclusive until Rosenbaum et al. [12] first attempted to build a molecular phylogeny that demonstrated *Eubalaena* should be differentiated from *Balaena* at the genus level (an authoritative book on marine mammal taxonomy published in 1998 [28] suggested that *Eubalaena* as a genus should be disregarded) as well as three distinct *Eubalaena* lineages. Now, three living species of *Eubalaena*, including *E. australis*, *E. glacialis*, and *E. japonica*, are widely accepted taxonomically [29, 30], but detailed morphological differences among *Eubalaena* spp. remain unresolved, as phylogenetic or morphological studies of baleen whales often treated *Eubalaena* spp. as a single operational taxonomical unit [6, 31, 32]. Baleen whale ear bones prove to be diagnostically and phylogenetically informative [6, 7, 19]. Thus, even only partially preserved, further research on the morphological disparity of the tympanic bulla of extant *Eubalaena* spp. may show that NTUM-VP 190807 (from the low latitude of the North Pacific) is morphologically closer either to *E. australis* (the Southern right whale) or to *E. japonica* (the North Pacific right whale), thus likely revealing the direction of dispersal. The discovery of NTUM-VP 190807 as a low-latitudinal occurrence of *Eubalaena* (Fig. 3, an artistic reconstruction) can be a starting point to decipher the direction and frequency of acquiring the antitropical distribution and should invite further research on the biotic interchange between two hemispheres.

Subtropical or even tropical occurrences of *Eubalaena* may not seem to be so unusual, because some distribution maps depict the range close to the Equator (Fig. 1, the proposed distribution of extant *Eubalaena* follows Wilson and Mittermeier [28]). However, three species of extant *Eubalaena* remain one of the best-known and most widely-cited examples of antitropical species pairs, indicating the preference for inhabiting the temperate waters in the Northern and Southern Hemispheres [29, 30]. Judging from the low latitudinal occurrence of *Eubalaena* represented by NTUM-VP 190807, this tropical balaenid likely existed on the southern margin of the western North Pacific during the glacial period; alternatively, if this specimen was Holocene in age, it might represent an extra-limital record during the interglaciation. The onset of glacial-interglacial shifts since the Pleistocene has long been associated with the antitropicality of marine mammals [2, 33]. In fact, closely related cetacean species (i.e., two distinct species within a genus) that demonstrate antitropical distributions can be found in the Mio/Pliocene (*Piscolithax* and *Messapicetus* [4, 34, 35]) and perhaps even Oligocene (*Yamatocetus*, [36]), making the link between the origin of antitropicality and climate oscillations in the Pleistocene not the sole explanation. Similarly, estimates of *Eubalaena* divergence varies in different studies (e.g., further back to the Pliocene or even Miocene [11, 13, 16, 32] or within the Pleistocene [14, 15]). Thus, NTUM-VP 190807 recovered from the Pleistocene (but see Locality and geological horizon for the age control) of Taiwan represents the first fossil evidence for supporting that the *Eubalaena* interchange between two hemispheres took place in the Pleistocene, most likely resulting from the glacial and interglacial periods that drove the distribution dynamics. Nevertheless, alternative interpretations may arise, such as extra-limital distribution, from future research on geological dating or recovery of additional fossils. In addition, the occurrence of NTUM-VP 190807 on the southern margin of the western North Pacific suggests that the western Pacific might be the corridor for the dispersal of *Eubalaena* when crossing the equator. If the western Pacific pathway indeed leads to the antitropical species pair of *Eubalaena*, it would, in turn, support the close phylogenetic relationship between northern *E. japonica* and southern *E. australis* [9–13], instead of a northern clade or the Atlantic-Southern pair [14–16]. Of note, *Eubalaena* interchanges between two hemispheres might have crossed the equator multiple times through various routes at different ages that resulted in phylogenetic inconsistency [9–17]; future finds should further reveal more details about the origin and evolutionary history of the antitropical distribution of *Eubalaena*.

Understanding the turnover of marine megafauna that gave rise to the modern biodiversity is crucial. Knowledge of the megafaunal evolution is not only vital to explaining how the ecological system and its service evolved, as marine megafauna are often nutrient transporters and reservoirs [37], but also to providing insights into the large-scale policymaking for the future, an effort to which conservation paleobiology seeks to contribute [38]. Taken the discovery of NTUM-VP 190807 together with recent finds in this decade, substantial effort has markedly improved our knowledge of marine megafauna in the Pleistocene. For example, *Caperea*, previously only known from the Southern Hemisphere, surprisingly occurred in the Northern Hemisphere as well [39]; *Herpetocetus*, once thought to exist only prior to the Pleistocene, unexpectedly survived well into the Pleistocene [40]; *Eschrichtius*, unable to recover its western Pacific population partly due to the unknown breeding site, likely used southern part of the Taiwan Strait for breeding and nursing calves [22]; and two extremely large species, blue and fin whales (*Balaenoptera musculus* and *B. physalus*), were first discovered and adequately documented from the Pleistocene [7, 41], further complicating their evolutionary history as *B. musculus* × *physalus* pair represents one of the most common hybridizations in marine mammals [42]. As climate change proceeds and unusual occurrences seem to happen more frequently [43–45], more effort into searching Pleistocene sediments, in both overlooked [46] or even well-sampled areas [47], should bring more surprises alive and guide us how to respond to global climate change.

## Conclusions

Discovery of a Pleistocene right whale from the low-latitudinal region (Taiwan) not only indicates that glacial and interglacial periods in the Pleistocene play a critical role in shaping the biological distribution, but also likely demonstrates that the western North Pacific used to be a corridor of biotic interchanges between two hemispheres. However, the geological dating of specimens from the sea bottom of Taiwan Strait remains poorly resolved, leading to the uncertainties of evolutionary and ecological interpretations; the effort to pin down the ages of various fossils or even Holocene remains should test the hypothesis of *Eubalaena* interchange presented in this paper. Further research into uncovering more Pleistocene fossils should reveal how biodiversity experienced origination, extinction, survivorship, and dispersal, etc. that leaded to the emergence of modern biodiversity.

## Data Availability

The original fossil is curated in the Lab of Evolution and Diversity of Fossil Vertebrates, Museum of Zoology, National Taiwan University. In addition, the 3D data of the actual fossil can be freely downloaded at: doi.org/10.5281/zenodo.3402015 or https://scholars.lib.ntu.edu.tw/handle/123456789/424590.
